# Inhibition of ANO1/TMEM16A induces apoptosis in human prostate carcinoma cells by activating TNF-α signaling

**DOI:** 10.1038/s41419-018-0735-2

**Published:** 2018-06-13

**Authors:** Yan Song, Jian Gao, Lizhao Guan, Xiaoling Chen, Jianjun Gao, KeWei Wang

**Affiliations:** 1 Department of Molecular and Cellular Pharmacology, State Key Laboratory of Natural and Biomimetic Drugs, Peking University School of Pharmaceutical Sciences, Beijing, 100191 China; 2 Department of Pharmacology, Qingdao University School of Pharmacy, Qingdao, 266021 China

## Abstract

Overexpression of the Ca^2+^-activated chloride channel ANO1/TMEM16A is implicated in tumorigenesis, and inhibition of ANO1 overexpression suppresses xenograft tumor growth and invasiveness. However, the underlying molecular mechanism for ANO1 inhibition in suppression of tumorigenesis remains unknown. Here, we show that silencing or inhibition of endogenous ANO1 inhibits cell growth, induces apoptosis and upregulates TNF-α expression in prostate cancer PC-3 cells. Enhancement of TNF-α signaling by ANO1 knockdown leads to upregulation of phosphorylated Fas-associated protein with death domain and caspase activation. Furthermore, silencing of ANO1 inhibits growth of PC-3 xenograft tumors in nude mice and induces apoptosis in tumors via upregulation of TNF-α signaling. Taken together, our findings provide mechanistic insight into promoting apoptosis in prostate cancer cells by ANO1 inhibition through upregulation of TNF-α signaling.

## Introduction

Prostate cancer originates from the glandular epithelial cells, and is one of the most common malignancies and the second leading cause of cancer-related death in males worldwide^1^. However, the pathogenesis of prostate cancer remains to be clearly defined. Ca^2+^-activated Cl^-^ channel anoctamin-1 (ANO1), also known as transmembrane member 16A (TMEM16A), is expressed in epithelial cells where it plays important roles in mediating chloride secretion for numerous physiological functions such as regulation of excitability in neurons and water–electrolyte balance^2,3^. ANO1 overexpression is involved in the tumorigenesis of epithelial cancers including oral cancer^4^, gastrointestinal stromal tumor (GIST)^5^, head and neck squamous cell carcinoma (HNSCC)^6^, prostate cancer^7^ and hyperplasia^8^, breast cancer^9^, colorectal cancer^10^, glioma^11^, esophageal squamous cell carcinoma^12^, pancreatic ductal adenocarcinoma^13^, lung cancer^14^, and hepatocellular carcinoma^15^.

*ANO1* gene is located within the chromosome 11q13 that is one of the most frequently amplified regions in human cancer and associated with poor prognosis^16–19^. ANO1 amplification and overexpression contribute to tumor growth by activating EGF receptor and calmodulin-dependent-protein kinase II, and subsequently enhancing AKT and mitogen-activated protein kinase (MAPK) signaling^9,20^. Silencing or inhibition of ANO1 suppresses proliferation, metastasis, and invasion of cancer cells^7,14,21–23^, and also promotes GIST cells to undergo apoptosis^24^. However, how ANO1 inhibition exerts anti-tumor activity or causes apoptosis in cancer cells remains unknown.

Apoptosis is a highly regulated cellular process critical for cell growth and tissue development^25^. Loss of apoptosis can lead to tumor initiation, growth, and progression^26^. Apoptosis is activated by intracellular mitochondrial signals (intrinsic pathway) and extracellular death ligands (extrinsic pathway) via death-inducing signaling complex (DISC)^27,28^. The DISC is composed of death receptor, FADD and caspase-8, transducing a downstream signal cascade resulting in apoptosis^28^. The Fas-associated protein with death domain (FADD), encoded by the *FADD* gene, is an adaptor protein that connects members of the tumor necrosis factor (TNF) receptor superfamily, such as Fas (TNF receptor superfamily, member 6), TRAIL-R (Tumor necrosis factor related apoptosis inducing ligand receptor), and TNFR1 (Tumor necrosis factor α receptor 1) to procaspases-8 to form the DISC, thus activating the cysteine protease cascade and inducing apoptosis^28^.

The cell signaling effect of TNF-α is primarily mediated by its receptor TNFR1^29,30^. TNFR1 is expressed in many tissues, and it initiates the majority of TNF-induced biological activities, including induction of cell death^30^. Binding of TNF-α to TNFR1 triggers a series of intracellular events, including caspase family-mediated apoptosis, the activation of NF-κB and c-Jun amino-terminal kinase (JNK) due to the formation of two separate complexes^31^. Complex 1 that mediates NF-κB induction is initiated through the recruitment of TNF receptor-associated protein with a death domain (TRADD). Complex 2 primarily mediated through FADD and caspase-8 activation activates the apoptotic pathway^31^.

Gene profiling of tumors by meta-analyses from microarray data sets shows that ANO1 and FADD, both located on chromosome 11q13, can serve as prognostic markers for breast cancer and head and neck cancer^32,33^, indicating a critical role of ANO1 in FADD-mediated apoptosis. Based on the literature reports and our previous findings, we therefore hypothesized that suppression of ANO1 overexpression may result in an upregulation of death receptor-ligand systems such as TNF-α signaling mediated by FADD, thus leading to suppression of tumor proliferation and metastasis. To test this hypothesis, we utilized genetic and pharmacological approaches to investigate the ANO1 expression and TNF-α signaling in prostate cancer cells. Our findings show that ANO1 expression in prostate cancer cells is negatively correlated with TNF-α signaling upstream to activation of caspase. Suppression of ANO1 upregulates TNF-α expression and activates TNF-α signaling, thus promoting apoptosis in prostate carcinoma.

## Results

### Suppression of ANO1 overexpression inhibits cell growth and induces apoptosis in prostate cancer PC-3 cells

**Fig. 1:**
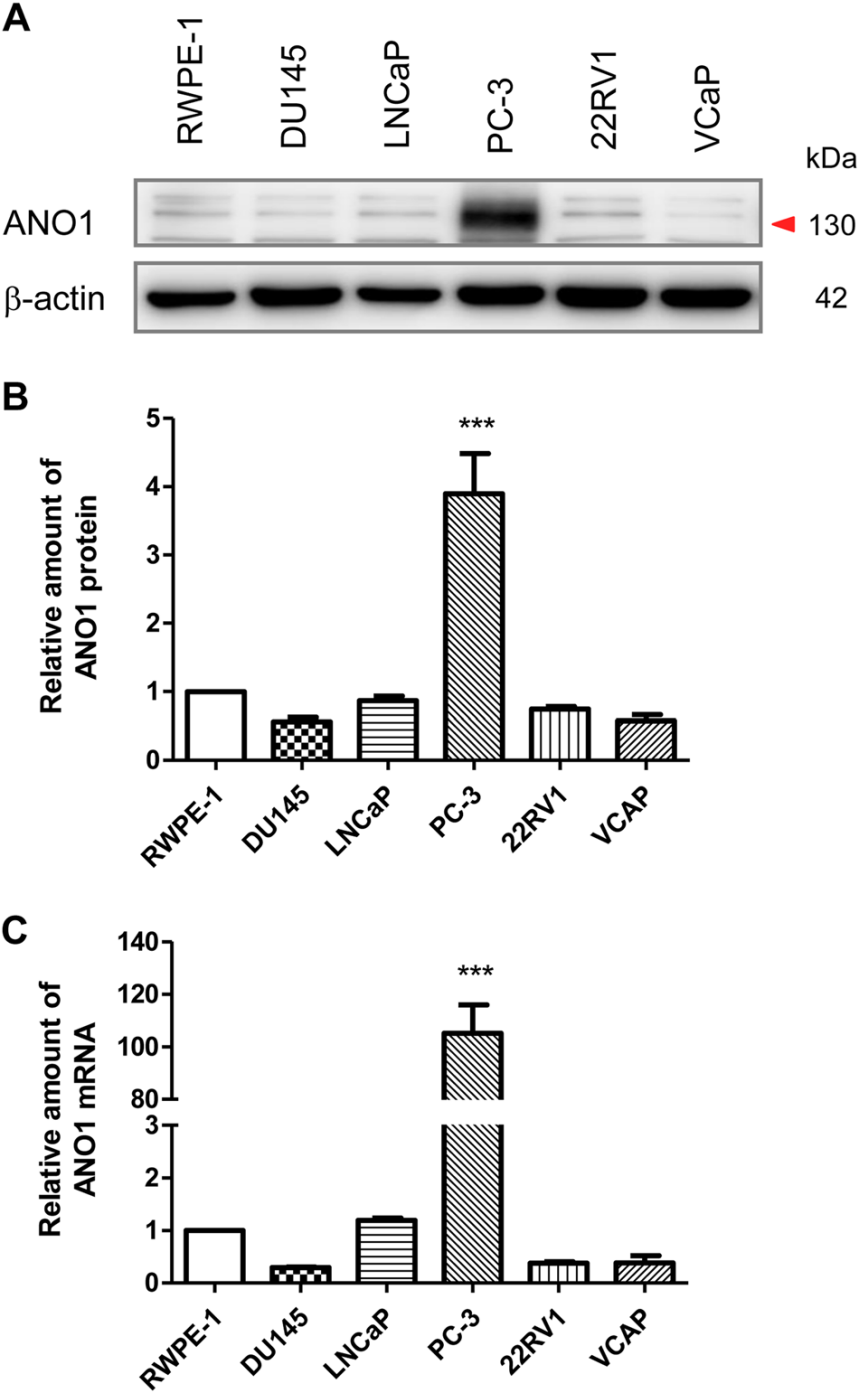
Comparison of ANO1 expression levels in different prostate cancer cell lines. **a** Immunoblot analysis of endogenous ANO1 expression in normal prostate epithelial cells (RWPE-1) and prostate cancer cell lines (DU145, LNCaP, PC-3, 22RV1, and VCaP) by western blot. The expression of ANO1 protein was normalized to the expression level of β-actin. **b** Quantitative analysis of ANO1 protein expression in cell lines from **a**. The ANO1 protein level is increased fourfold in PC-3 cells, as compared with that of RWPE-1 cells. **c** Quantitative real time PCR analysis of ANO1 mRNA levels in RWPE-1, DU145, LNCaP, PC-3, 22RV1, and VCaP cells. The ANO1 mRNA level is increased 100-fold in PC-3 cells, as compared with that of RWPE-1 cells. All data are shown as the means ± SEM (*n* *=* 4 independent experiments). The statistical significance between groups is indicated as *** *p* < 0.001

To investigate the biological function of ANO1, we compared the protein and mRNA levels of ANO1 in normal prostate epithelial cells (RWPE-1) and prostate cancer cell lines (DU145, LNCaP, 22RV1, VCaP, and PC-3). Among all the cell lines, PC-3 cells expressed markedly higher levels of ANO1 proteins and mRNAs (Fig. 1). Compared with RWPE-1 cells, ANO1 protein expression in PC-3 cells was about fourfold higher (Fig. 1b), and ANO1 mRNA expression in PC-3 cells also increased by >100-fold (Fig. 1c).

**Fig. 2:**
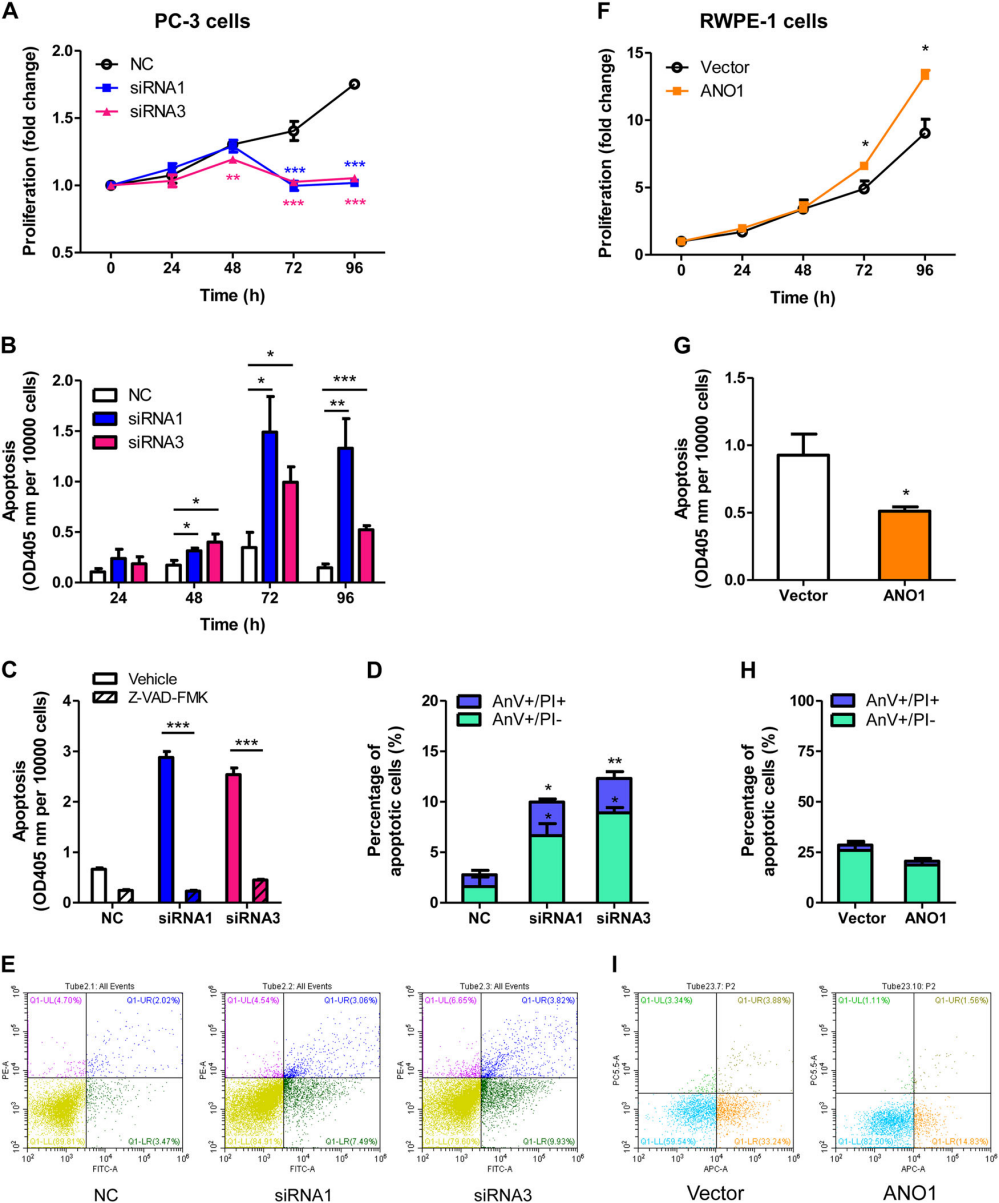
Inhibition of ANO1 expression suppresses cell growth and induces apoptosis in prostate cancer cells. **a–e** Silencing of endogenous ANO1 decreases prostate cancer cell growth and induces apoptosis in PC-3 cells. **a** PC-3 cells were transfected with ANO1-siRNAs or NCsi (negative control scrambled siRNA), and viable cell number was accessed by CCK-8 assay (means ± SEM, *n* = 6). **p *<* 0.01; ***p *<* 0.001 vs NC (negative control). **b** Bar graph showing apoptosis accessed by Cell Death Detection ELISA^PLUS^ Kit (means ± SEM, *n* = 4). Parallel plates with the same treatment were used for cell counting. **c** PC-3 cells were transfected with ANO1-siRNAs for 72 h and co-treated with 30 μM of Z-VAD-FMK for 48 h. Z-VAD-FMK, a pan-caspase inhibitor, blocks apoptosis induced by ANO1 silencing (means ± SEM, *n* = 4). ***p *<* 0.001 vs vehicle (0.1% DMSO). **d** Bar graph showing percentage of apoptotic cells after knockdown of ANO1 for 72 h (means ± SEM, *n* = 3). Apoptosis was assessed by Annexin V-FITC/PI Apoptosis Detection Kit using a Flow Cytometer. **e** Representative cytometry images of PC-3 cells treated with ANO1-siRNAs or NCsi. **f** ANO1 overexpression increases RWPE-1 cell growth. ANO1 was stably overexpressed in human normal prostate epithelial RWPE-1 cells by transfection of ANO1 plasmid in pIRES2-EGFP vectors. Data are presented as the means ± SEM; *n* = 3 independent experiments. *p *<* 0.05 vs vector. **g–i** ANO1 overexpression inhibits RWPE-1 cell apoptosis accessed by Cell Death Detection ELISA^PLUS^ Kit (**g**: *n* = 4) or Annexin V-APC/7-ADD Apoptosis Detection Kit (**h**: *n* = 3)

To verify the causal role of ANO1 in the pathogenesis of prostate cancer, two siRNAs were used to knockdown ANO1 expression in prostate cancer PC-3 cells. Both ANO1-siRNAs significantly reduced ANO1 mRNA and protein expressions by ~90% and ~80%, respectively (Supplementary Figure 1). Knockdown of ANO1 resulted in significant reduction of cell proliferation in a time-dependent manner as compared with the scrambled siRNA as negative control (NC) (Fig. 2a). Cell death analysis revealed that ANO1 silencing induced marked apoptosis, which could be blocked with a pan-caspase inhibitor (Z-VAD-FMK^34^, Fig. 2c). However, silencing ANO1 does not significantly decrease cell proliferation and induce apoptosis in RWPE-1 or DU145 cells with ANO1 lower expression (Supplementary Figure 2). To confirm the effect of ANO1 silencing on cell growth and apoptosis, we exogenously transfected ANO1 complementary DNA (cDNA) into normal epithelial RWPE-1 cells to overexpress 1500 folds of ANO1 mRNAs and 30 folds of ANO1 proteins (Supplementary Figure 3). By contrast, overexpressing ANO1 in RWPE-1 cells promoted cell proliferation (Fig. 2f) and inhibited apoptosis (Fig. 2g–i).

**Fig. 3:**
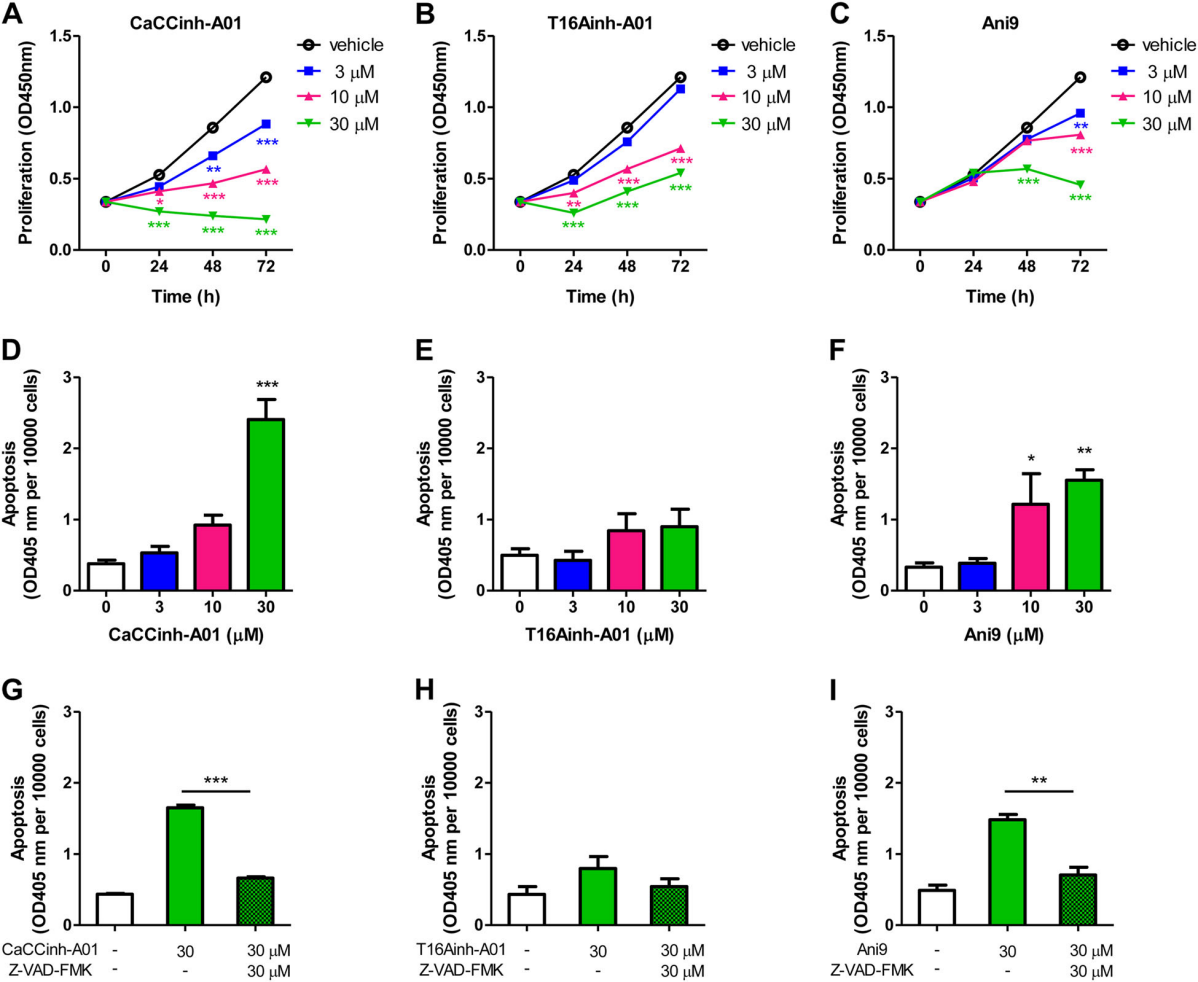
Pharmacological inhibition of ANO1 decreases cell growth and induces apoptosis in PC-3 cells. **a–c** PC-3 cells were treated with different concentrations of ANO1 inhibitors CaCCinh-A01 (**a**), T16Ainh-A01 (**b**), or Ani9 (**c**) in 0.5% FBS medium. Cell proliferation was measured by CCK-8 assay. Data are presented as the means ± SEM; *n* = 6. *p < 0.05, **p < 0.01, ***p < 0.001 vs vehicle (0.1% DMSO). Inhibition of ANO1 with CaCCinh-A01, T16Ainh-A01, or Ani9 decrease viable cell number in a dose-dependent or time-dependent manner. **d–f** Bar graphs showing apoptosis of PC-3 cells after 72 h inhibition of ANO1 with CaCCinh-A01 (**d**), T16Ainh-A01 (**e**), or Ani9 (**f**) (means ± SEM; *n* = 4). *p *<* 0.05, **p *<* 0.01, ***p *<* 0.001 vs vehicle (0.1% DMSO). CaCCinh-A01 and Ani9 markedly induce apoptosis in a good dose-dependent manner whereas T16Ainh-A01 shows the weak effect. **g**–**i** Z-VAD-FMK, a pan-caspase inhibitor, blocks apoptosis induced by CaCCinh-A01 and Ani9. PC-3 cells were treated with CaCCinh-A01 (**g**), T16Ainh-A01 (**h**), or Ani9 (**i**) and co-treated with 30 μM of Z-VAD-FMK for 72 h. Data are expressed as the means ± SEM (*n* = 4). **p *<* 0.01, ***p *<* 0.001 for statistical significance and comparisons between Z-VAD-FMK versus vehicle groups

Given that ANO1 knockdown inhibited cell growth and induced apoptosis, we further tested the effects of three ANO1 inhibitors, CaCCinh-A01^35^, T16Ainh-A01^36^, and Ani9^37^ on PC-3 cells. All three compounds inhibited cell growth in dose-dependent or time-dependent manner (Fig. 3a–c). CaCCinh-A01 (3–30 μM) also induced apoptosis in concentration-dependent manner with about sixfold increase in apoptosis at the concentration of 30 μM, as compared with the vehicle treated cells (Fig. 3d). Z-VAD-FMK (30 μM), a pan-caspase inhibitor, markedly blocked apoptosis induced by CaCCinh-A01 (Fig. 3g) or Ani9 (Fig. 3i). These results indicated that ANO1 overexpression promoted the growth of prostate cancer PC-3 cells or normal RWPE-1 cells, whereas suppressing ANO1 induced apoptosis in PC-3 cells.

### Suppression of ANO1 enhances TNF-α expression and secretion

To understand the mechanism underlying the apoptosis induced by ANO1 inhibition, we performed the gene profiling analysis in prostate cancer PC-3 cells and found that ANO1 downregulation caused an increased expression of death receptor signaling molecules, such as TNF-α, TNFSF10 (TRAIL), and CASP7 (Supplementary Table 1 and Supplementary Figure 4). This result allowed us to hypothesize that silencing ANO1 might cause apoptosis in prostate cancer cells via enhancement of TNF-α signaling that in turn leads to the suppression of tumor proliferation and metastasis.

**Fig. 4:**
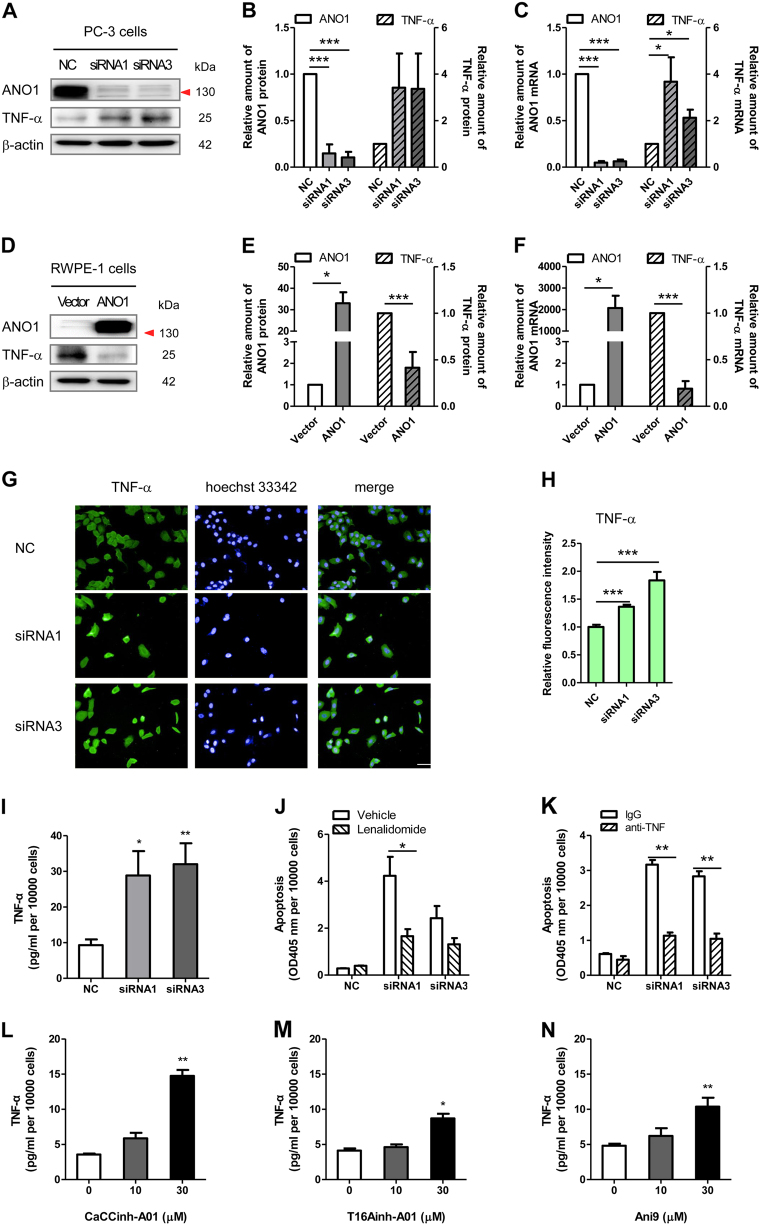
TNF-α expression is inversely correlated with ANO1 expression in prostate cancer cells. **a–c** Silencing of endogenous ANO1 increases TNF-α expression in PC-3 cells. **a** Representative immunoblots of ANO1 suppression by siRNAs and TNF-α upregulation. **b** Quantitative analysis of ANO1 and TNF-α protein expression from (**a**) (means ± SEM, *n* = 3). **c** Relative mRNA expressions of ANO1 and TNF-α was examined using qPCR and data are presented after being normalized to β-actin (means ± SEM; *n* = 4). * *p* *<* 0.05; ** *p* *<* 0.01; *** *p* *<* 0.001 vs NC. **d–f** Normal prostate epithelium RWPE-1 cells stably transfected with ANO1 show a decrease of TNF-α protein and mRNA expression. Data are expressed as the means ± SEM (**e** *n* = 3; F: *n* = 4). * *p* *<* 0.05; ** *p* *<* 0.01 vs vector. **g** Immunostaining images in PC-3 cells treated with ANO1-siRNAs or NCsi for 72 h (magnification × 20). **h** Bar graph showing quantitative analysis of fluorescence intensity of TNF-α from (**g**). Data are presented as the means ± SEM; *n* = 4; ***p *<* 0.001 vs NC. **i** Silencing of endogenous ANO1 increases TNF-α production in PC-3 cells. Levels of TNF-α in culture supernatants were measured by ELISA. Parallel plates with the same treatment were used for cell counting. Data are expressed as the means ± SEM (*n* = 4). *p *<* 0.05; **p *<* 0.01 vs NC. **j** Lenalidomide reverses apoptosis induced by silencing ANO1. PC-3 cells were transfected with ANO1-siRNAs for 72 h and co-treated with 20 nM of lenalidomide for 24 h. Data are expressed as the means ± SEM (*n* = 4). * *p* *<* 0.05, **p *<* 0.01 for statistical significance for comparisons between lenalidomide versus vehicle groups. **k** Human TNF-α antibody reduces apoptosis induced by silencing ANO1. PC-3 cells were transfected with ANO1-siRNAs for 72 h and co-treated with 0.2 μg/ml of anti-TNF or normal IgG for 48 h. Data are expressed as the means ± SEM (*n* = 4). ** *p* *<* 0.01 for statistical significance for comparisons between human TNF-α antibody versus normal goat IgG. **l–n** ANO1 inhibitors, CaCCinh-A01 (**l)**, T16Ainh-A01 (**m**), or Ani9 (**n**) increased TNF-α production in PC-3 cells. Levels of TNF-α in culture supernatants were measured by ELISA. Parallel plates with the same treatment were used for cell counting. Data are normalized and expressed as the means ± SEM (*n* = 4). *p *<* 0.05; **p *<* 0.01 vs control (0.1% DMSO)

To investigate the correlation between ANO1 suppression and TNF-α upregulation, we determined the effect of silencing ANO1 on TNF-α expression in PC-3 cells. Silencing of ANO1 by either siRNA1 or siRNA3 resulted in approximate threefold elevation of TNF-α protein expression in PC-3 cells (Fig. 4a, b), which was consistent with the real time quantitative PCR analysis (Fig. 4c). We also overexpressed ANO1 and examined TNF-α protein and mRNA expressions in normal RWPE-1 cells that express a low level of ANO1 and a robust TNF-α level. As expected, ANO1 overexpression caused a decrease of TNF-α mRNA and protein expressions in RWPE-1 cells to 41 ± 17% (Fig. 4e) and 18 ± 8% (Fig. 4f), respectively. TNF-α level in PC-3 cells was further evaluated by immunostaining. Transfection with ANO1-siRNAs in PC-3 cells caused a significant increase of TNF-α fluorescence intensity, as compared with negative control cells (Fig. 4g, h). We also determined TNF-α secretion level in culture supernatant. Silencing of ANO1 resulted in about threefold elevation of TNF-α secretion in PC-3 cells (Fig. 4i). These results showed that silencing of endogenous ANO1 inversely increased TNF-α expression and secretion in PC-3 cells.

To further investigate the role for TNF-α in ANO1 silencing-induced apoptosis, we used lenalidomide, an immunomodulatory agent inhibiting TNF-α production^38,39^, and TNF-α neutralizing antibody^40^. Both lenalidomide at 20 nM (Fig. 4j) and human TNF-α neutralizing antibody at 0.2 μg/ml (Fig. 4k) reduced ANO1 silencing-induced apoptosis in PC-3 cells, demonstrating that ANO1 silencing-induced apoptosis is TNF-α-dependent.

To test the impact of pharmacological inhibition of ANO1 on TNF-α secretion in PC-3 cells, we utilized three ANO1 inhibitors CaCCinh-A01, T16Ainh-A01, and Ani9. Addition of individual ANO1 inhibitor resulted in an increase of TNF-α secretion detected by enzyme linked immunosorbent assay (ELISA) in dose-dependent manner (Fig. 4l–n). In addition, we also determined the effect of ANO1 activation by compound Eact on classical lipopolysaccharide (LPS)-stimulated TNF-α secretion in primary mouse macrophages^41^. Eact, a selective ANO1 activator with EC_50_ between 3 and 6 μM^42^, suppressed TNF-α secretion in LPS-stimulated mouse macrophages in dose-dependent manner (Supplementary Figure 5).

Having observed the suppression of TNF-α expression by ANO1 activation, we further examined the effect of TNF-α on ANO1 expression in PC-3 cells treated with different concentrations of human recombinant TNF-α (10 pg/ml, 10 ng/ml, and 10 μg/ml). The result showed that TNF-α caused the downregulation of ANO1 protein and mRNA expression in PC-3 cells (Supplementary Figure 6), further confirming the inverse correlation between ANO1 and TNF-α expression.

### Silencing ANO1 promotes TNF-α downstream signaling by phosphorylating FADD and activating caspase family

**Fig. 5:**
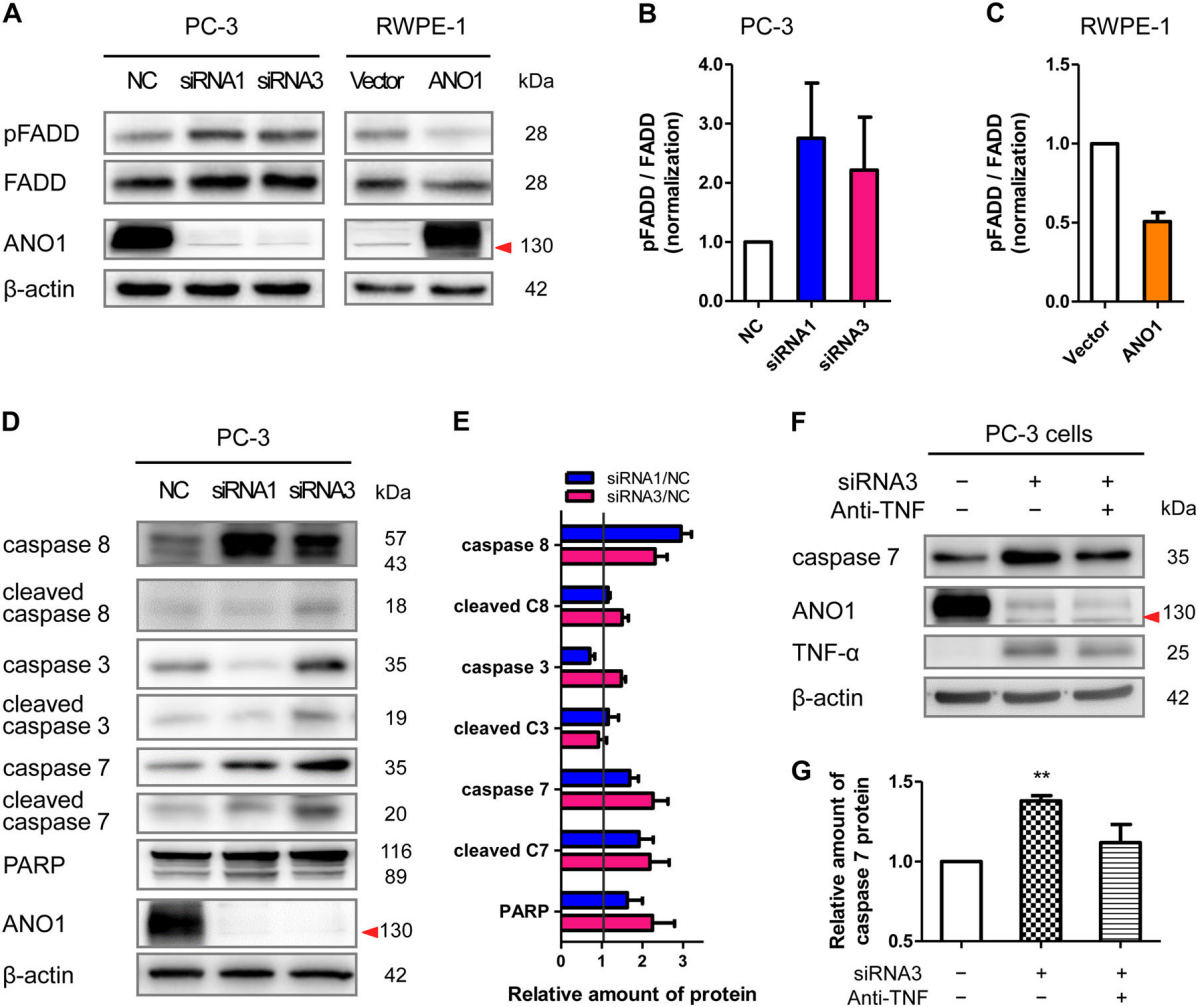
Silencing ANO1 activates TNF-α downstream signaling by phosphorylation of FADD and activation of caspase family members for induction of apoptosis. **a**–**c** Silencing of endogenous ANO1 promotes phosphorylation of FADD. Left panel of (**a**), Immunoblots of lysates from PC-3 cells transfected with ANO1-siRNAs or NCsi for 72 h. Right panel of (**a**), Immunoblots of lysates from normal RWPE-1 cells stably expressing ANO1 or the IRES2-GFP vector as the control. Bar graph showing quantitative analysis of phospho-FADD and total FADD protein expression in PC-3 cells with ANO1 knockdown (**b**: *n* = 4) and RWPE-1 cells with ANO1 overexpression (**c**: *n* = 3) from (**a**). **d**, **e** Silencing of endogenous ANO1 promotes the expression of caspases. Immunoblots of lysates from PC-3 cells transfected with ANO1-siRNAs or NCsi for 72 h. Bar graph showing quantitative analysis of protein expressions (**e** *n* = 4) from (**d**). Data were normalized to NC group cells. **f**, **g** Human TNF-α antibody reduces caspase-7 overexpression induced by ANO1 knockdown in PC-3 cells. Bar graph showing quantitative analysis of caspase-7 protein expressions (**g** *n* = 4, **p *<* 0.01 vs NC) from (**f**)

To investigate the effect of ANO1 silencing-induced TNF-α upregulation on its downstream signaling, we analyzed the effects of ANO1 knockdown or overexpression on expression of TNF-α signaling proteins such as TNFR1, TRADD, phosphorylated-FADD (pFADD), and FADD by western blot. As shown in Fig. 5a, ANO1 knockdown significantly increased pFADD level in PC-3 cells. In contrast, ANO1 overexpression suppressed pFADD level in normal RWPE-1 cells. As a control, the expression of TNFR1 and TRADD was not significantly different in either PC-3 cells or RWPE-1 cells (Supplementary Figure 7). These results suggested that ANO1 knockdown promoted the phosphorylation of FADD and formation of complex 2, subsequently leading to the induction of cell apoptosis.

When the complex 2 is formed, caspase-8 is activated, resulting in the cleavage of its downstream substrates such as caspase-3 and -7 toward apoptosis execution^28^. We therefore examined the protein expression and activation of the caspases. As shown in Fig. 5d, e, ANO1 knockdown significantly increased protease expression and activation of caspase-8, caspase-3, caspase-7, and PARP (poly ADP-ribose polymerase) in prostate cancer PC-3 cells. Furthermore, TNF-α antibody caused a reduction of ANO1 silencing-increased caspase-7 expression in PC-3 cells (Fig. 5f, g). These results indicated that silencing ANO1 promoted TNF-α downstream signaling such as phosphorylation of FADD, activation of caspase family members, thus leading to apoptosis in prostate cancer PC-3 cells.

**Fig. 6:**
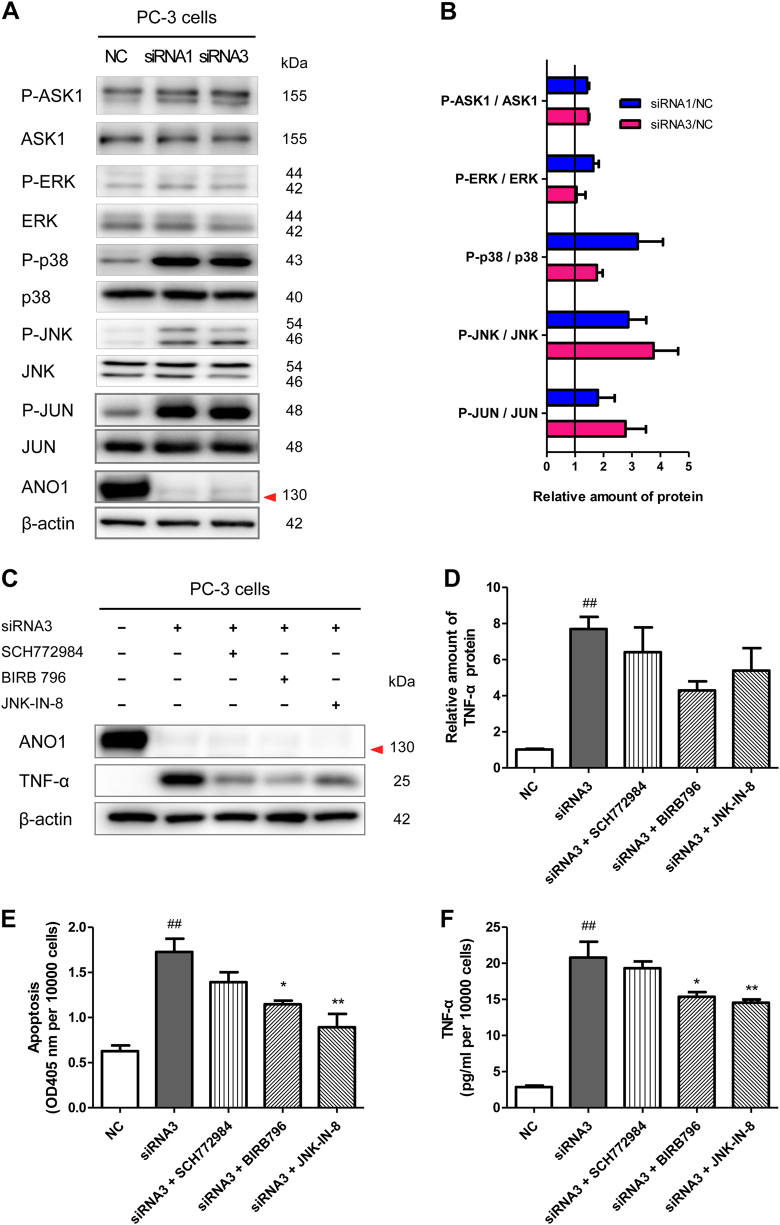
Silencing ANO1 upregulates TNF-α expression through MAPK signaling. **a** Silencing of endogenous ANO1 promotes phosphorylation of ASK1, p38, JNK, and JUN. Immunoblots of lysates from prostate cancer PC-3 cells transfected with ANO1-siRNAs or NCsi for 72 h. **b** Bar graph showing quantitative analysis of protein expression from (**a**). Data were normalized to NC group cells (means ± SEM; *n* = 4). **c** MAPK inhibitors inhibit upregulation of TNF-α induced by ANO1 silencing. Immunoblots of lysates from PC-3 cells transfected by ANO1-siRNA3 or NCsi for 72 h and co-treated with 0.3 μM SCH772984 (ERK inhibitor), BIRB 796 (p38 inhibitor), or JNK-IN-8 (JNK inhibitor) for 24 h, respectively. **d** Bar graph showing quantitative analysis of protein expression from (**c**). Data are presented as the means ± SEM (*n* = 3). ^##^*p* *<* 0.01 vs NC group. **e**, **f** BIRB 796 and JNK-IN-8 reverse the enhancement of TNF-α expression and apoptosis induced by ANO1 silencing. PC-3 cells were transfected by ANO1-siRNA3 or NCsi for 72 h and co-treated with 0.3 μM SCH772984, BIRB 796, or JNK-IN-8 for 24 h, respectively. **e** Apoptosis was assessed by Cell Death Detection ELISA^PLUS^ Kit. **f** Levels of TNF-α in culture supernatants were measured by ELISA. Data are presented as the means ± SEM (*n* = 5). ^##^*p* *<* 0.01 vs NC group. *p *<* 0.05, **p *<* 0.01 vs siRNA3 group

To understand how ANO1 knockdown increased TNF-α expression, we examined the MAPK signaling that has been reported to regulate TNF-α expression through p38α that plays a key role in control of TNF-α production^43,44^. ANO1 knockdown significantly elevated the levels of phosphorylated ASK1 (Apoptosis signal-regulating kinase 1), p38, JNK, and JUN (Jun proto-oncogene) in PC-3 cells (Fig. 6a, b). Both p38 inhibitor BIRB 796 (0.3 μM) and JNK inhibitor JNK-IN-8 (0.3 μM) reversed TNF-α upregulation (Fig. 6c–f) and apoptosis (Fig. 6e) induced by ANO1 silencing. These results suggested that ANO1 knockdown likely promoted the phosphorylation of p38 and JNK, and consequently increases TNF-α production.

### Silencing of ANO1 inhibits xenograft tumor growth in nude mice

**Fig. 7:**
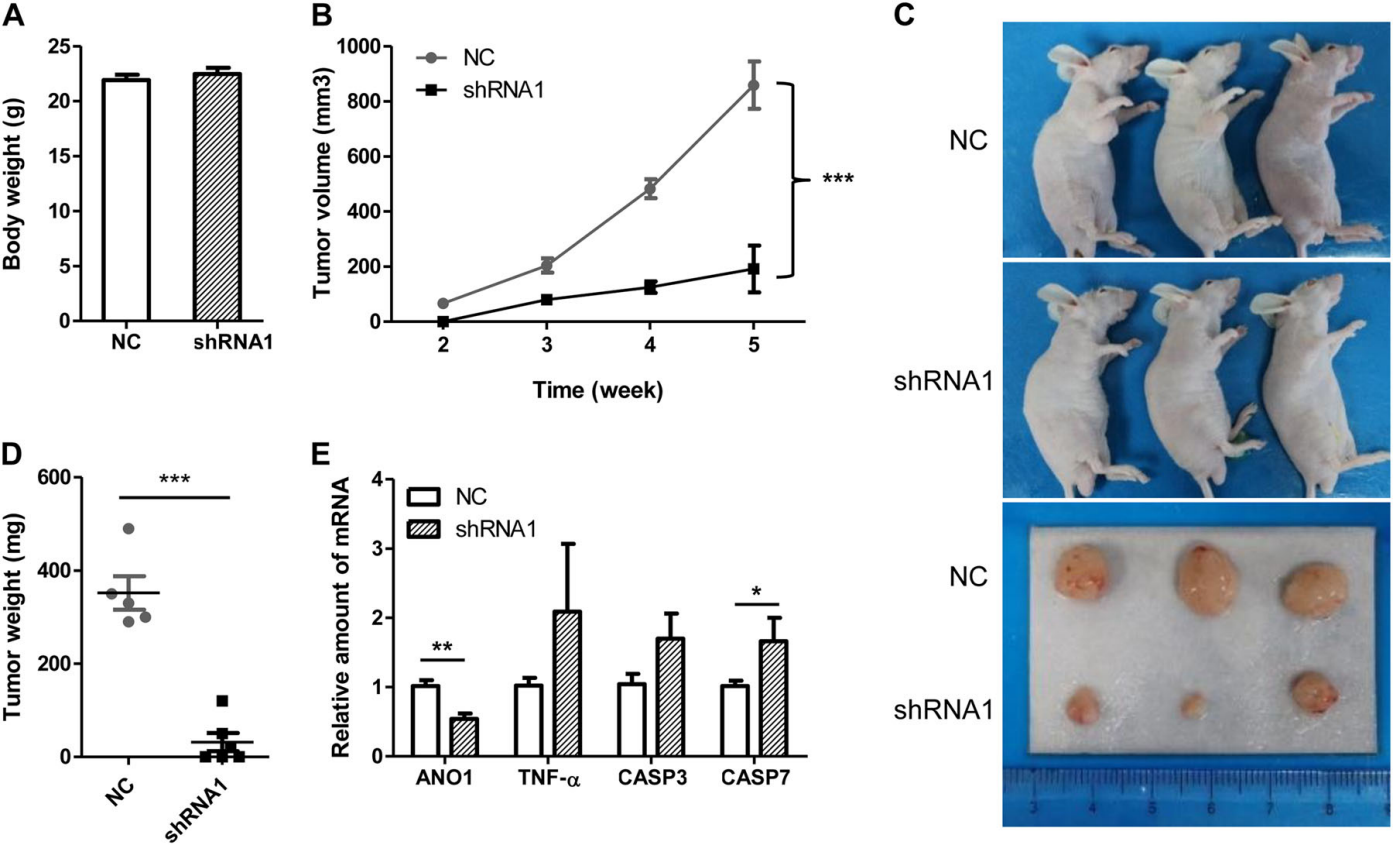
Silencing of ANO1 inhibits xenograft tumor growth in nude mice by activating TNF-α signaling. **a** Body weight in nude mice. The lentiviral vector pLKO.1 was used to construct ANO1 shRNA1 and control shRNA plasmids. In total, 2 × 10^6^ PC-3 cells with lentiviral infection of ANO1 shRNA1 or NCsh (negative control scrambled shRNA) were injected into the right flank of nude mice of the same age. **b** Knockdown of ANO1 resulted in significant inhibition of xenograft tumor growth. Tumor volumes in each group were measured with calipers once a week. The data are shown as the average tumor volume in mm^3^ ± SEM (NC, *n* = 5; shRNA1, *n* = 6). ***p *<* 0.001 vs NC. **c** Comparisons of tumor bearing mice and tumors that were removed on the 35th day from groups of negative control and ANO1 siRNA1. **d** Tumor weights in nude mice between groups of negative control (NC, 352 ± 36 mg, *n* = 5) and ANO1 siRNA1 (shRNA1, 63 ± 30 mg, *n* = 6). ***p *<* 0.001 vs NC. **e** Silencing ANO1 increased the mRNA expression of TNF-α, CASP3, and CASP7 in xenograft tumors. The relative mRNA expressions were examined using qPCR and presented after being normalized to β-actin (means ± SEM; NC, *n* = 5; shRNA1, *n* = 3. *p *<* 0.05; **p *<* 0.01 vs NC)

To further confirm the role for ANO1 knockdown in tumor growth in vivo, we constructed lentiviral vectors expressing ANO1 shRNA1 for silencing ANO1 expression in PC-3 cells as reported in our previous investigations^23^. Three days after lentiviral infections, ~ 2 × 10^6^ PC-3 cells treated with ANO1 shRNA1 or NC shRNA were injected into the right flank of nude mice of the same age for formation of xenograft tumors. Body weight and tumor volumes were measured every week during the period of observation until the animals were sacrificed. During the experiment, no animal death and signs of toxicity were observed. There was also no significant difference between body weights in ANO1 shRNA1 mice and NC shRNA mice (Fig. 7a).

As shown in Fig. 7b, the average tumor volume in control mouse group was ~ 859 ± 86 mm^3^ (*n* = 5) at the termination of experiment. By contrast, treatment with ANO1 shRNA1 resulted in a significant reduction in tumor volume, which was 96 ± 57 mm^3^ (*n* = 6), ~ 11% of the tumor volume in the control group. The average weight of tumors in ANO1 shRNA1 group (32 ± 19 mg, *n* = 6) was significantly reduced, as compared with the NC group (352 ± 36 mg, *n* = 5) (Fig. 7c, d). These results demonstrated that silencing of ANO1 inhibited the growth of xenograft tumor in nude mice.

To examine whether knockdown of ANO1 altered apoptotic signaling, we determined gene expression in the tumor xenografts. As compared with the NC tumors, ANO1 mRNA expression was suppressed by ~ 53.9 ± 8.1% in xenograft tumors treated with ANO1 shRNA1 (*p* *<* 0.01, Fig. 7e). Silencing ANO1 also increased TNF-α, CASP3, and CASP7 mRNA expression by 2.1-fold, 1.7-fold, and 1.7-fold, respectively. These results further confirmed that ANO1 silencing induced apoptosis in PC-3 cell-derived prostate tumors.

## Discussion

**Fig. 8:**
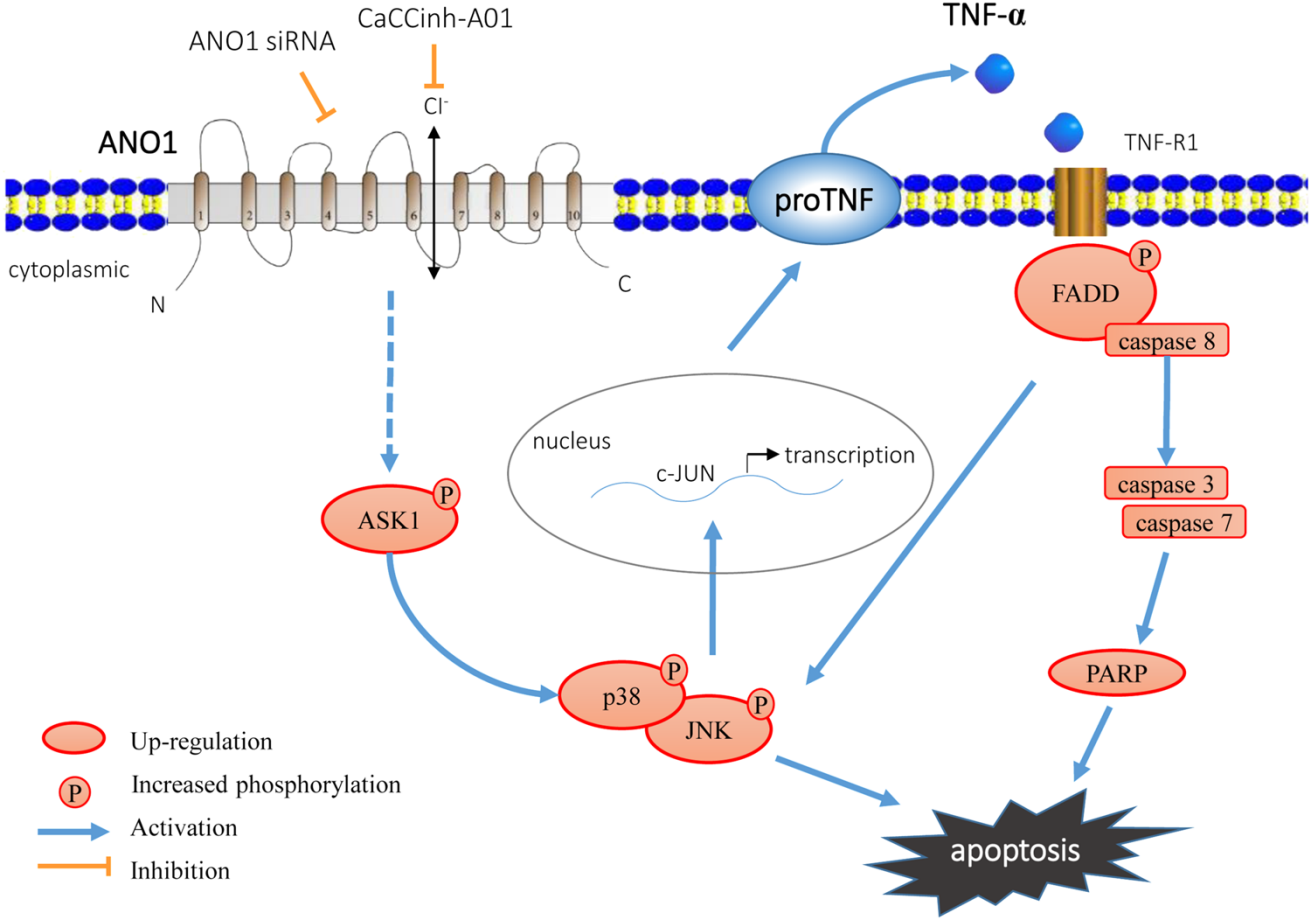
A proposed model for apoptosis induced by suppressing ANO1 through activation of TNF-α signaling in prostate cancer. Silencing or pharmacological inhibition of endogenous ANO1 upregulates TNF-α expression and promotes TNF-α signaling through phosphorylation of FADD (Fas-Associated protein with Death Domain), activation of caspase family, and activation of JNK (c-Jun N-terminal kinase) and JUN (Jun proto-oncogene), thus leading to the induction of apoptosis in prostate cancer cells. Silencing of ANO1 may also increase TNF-α production through MAPK (mitogen-activated protein kinase) signaling, such as phosphorylation of p38 and JNK, and inducing apoptosis in prostate cancer cells

We previously found that the expression of ANO1 in prostate cancer was upregulated and associated with the progression of cancer malignancy. Inhibition of ANO1 suppressed tumor cell proliferation and migration in vitro, and tumor growth in vivo^7^. The goal of this study was to investigate the molecular mechanism underlying ANO1 inhibition in suppression of prostate cancer cell growth. Based on the literature and our previous findings, we hypothesized that suppression of ANO1 results in enhancement of caspase upstream pathways such as TNF-α signaling, subsequently leading to inhibition of tumor proliferation and metastasis. To test this hypothesis, we utilized genetic and pharmacological approaches to investigate the effect of ANO1 expression on TNF-α signaling in prostate cancer cells. Based on our and others’s findings, we proposed that silencing or pharmacological inhibition of endogenous ANO1 upregulates TNF-α expression, and promotes TNF-α signaling cascade through phosphorylation of FADD, activation of caspase family, and activation of JNK and JUN, thus leading to induction of apoptosis in prostate cancer cells. Silencing of ANO1 can increase TNF-α production through MAPK signaling, such as activation of p38 and JNK (Fig. 8).

Our findings that silencing of endogenous ANO1 upregulates TNF-α expression in PC-3 cells, whereas stable overexpression of ANO1 downregulates TNF-α expression in normal RWPE-1 cells are consistent with the report that TMEM16A overexpression decreases the LPS-induced TNF-α secretions, and TMEM16A knockdown increases the LPS-induced TNF-α secretions in A549 cells^45^, supporting our view that ANO1 is inversely correlated with TNF-α expression. It still remains elusive how ANO1 silencing enhances expression of TNF-α. It has been reported that MAP kinase functions as both upstream and downstream of TNF-α signaling^43^. We think that binding of TNF-α to TNFR1 triggers a series of intracellular events, including the activation of JNK and p38. Activated JNK and p38 induce a secondary response by increasing the expression of several inflammatory cytokines (including TNF-α). Therefore, silencing of ANO1 promotes TNF-α signaling likely through the activation of p38 and JNK.

It is still debatable whether chloride channel activity of ANO1 is required for its pro-survival properties. Treatment with channel inhibitors abrogates cell proliferation in many cancer cell lines including breast cancer and HNSCC^46^. However, it is reported that inhibition of ANO1 activity alone is not sufficient to inhibit cancer cell proliferation, suggesting a novel function of ANO1 protein in cancer^47^. In this study, we found that ANO1 inhibitor CaCC_inh_-A01 and Ani9 can reduce cell viability and induce apoptosis by up-regulating TNF-α expression in a dose-dependent manner, whereas another inhibitor T16A_inh_-A01 shows weaker effect on cell proliferation and apoptosis. This is probably due to the downregulation of ANO1 expression by CaCC_inh_-A01 (30 μM)^23^, although the mechanism of action remains elusive.

In summary, our findings reveal a novel mechanism underlying the role of ANO1 inhibition in suppression of prostate cancer cells and xenograft tumors. ANO1 expression is inversely correlated with TNF-α expression in prostate cancer cells. Silencing or pharmacological inhibition of ANO1 upregulates TNF-α expression, which in turn promotes TNF-α signaling by enhancing phosphorylation of FADD, activating caspases and JNK and JUN. All these cascade events ultimately lead to the induction of apoptosis and inhibition of tumor growth. Therefore, suppressing ANO1 or enhancing TNF-α signaling may serve as a therapeutic strategy for potential treatment of human prostate cancer.

## Materials and methods

### Cell culture

Human prostate cancer PC-3, DU145, LNCaP, 22RV1 cells, and human normal prostate epithelial RWPE-1 cells were cultured in RPMI-1640 medium (Gibco, Grand Island, USA) supplemented with 10% fetal bovine serum (FBS; Gibco, Grand Island, USA). Human prostate cancer VCaP cells and human embryonic kidney 293LTV cells were maintained in DMEM medium (Gibco, Grand Island, USA) supplemented with 10% FBS. The cells were cultured at 37 °C with 5% CO_2_ in a humidified incubator. RWPE-1, DU145, and LNCaP cells were obtained from China Infrastructure of Cell Line Resources (Beijing, China). 22RV1 and VCaP cells were obtained from KeyGEN BioTECH Corp (Jiangsu, China). PC-3 and 293LTV cells were gifts from Dr. Hongquan Zhang at the Department of Anatomy, Histology and Embryology, Peking University Health Science Center. All cell lines were genotyped to establish their identities within the 6 months of experimentation.

### Chemicals

ANO1 inhibitors, CaCCinh-A01 (Tocris Bioscience, Bristol, UK), T16Ainh-A01 (Sigma, St. Louis, USA), Ani9 (Sigma, St. Louis, USA), and ANO1 opener Eact (Tocris Bioscience, Bristol, UK) were dissolved in dimethyl sulphoxide (DMSO) to a stock concentration of 30 mM. Z-VAD-FMK (pan-caspase inhibitor), Lenalidomide/CC-5013, SCH772984 (ERK inhibitor), Doramapimod (BIRB 796, p38 inhibitor), and JNK-IN-8 (JNK inhibitor) (Selleck, Houston, USA) were dissolved in DMSO to a stock concentration of 1 mM. Cells were treated with indicated concentrations of inhibitors or matching volumes of DMSO for control.

### Transfection with siRNA

ANO1/TMEM16A siRNAs and negative control siRNA were constructed by Ribobio Co., Ltd. (Guangzhou, China) according to the target sequences previously used in our laboratory^7^. siRNA1: CGTGTACAAAGGCCAAGTA (773–790 nt) and siRNA3: CGAAGAAGATGTACCACAT (533–550 nt). Transfection with siRNAs was carried out using Lipofectamine™ RNAiMAX reagent (Invitrogen, Carlsbad, USA) according to the manufacturer’s instructions. In all experiments with siRNA transfection, a missense siRNA was included as negative control.

### Establishment of ANO overexpression epithelial cell lines

The full-length cDNA for human *ANO1 (GenBank NM_018043)* was cloned into pIRES2-EGFP vector to construct ANO1 plasmids via standard molecular biological techniques. RWPE-1 cells were transfected with ANO1 plasmids or vector (negative control) using Lipofectamine™ 2000 reagent (Invitrogen, Carlsbad, USA) according to the manufacturer’s instructions. After G418 sulfate (800 μg/mL, Sigma, St. Louis, USA) screen, cells were identified using fluorescent microscope, qPCR, and western blot. The stable cell line that overexpressed exogenous ANO1 was successfully established and was used for cell viability and apoptosis evaluation.

### RNA extraction, reverse transcription, and qPCR analysis

Total RNAs were extracted using Trizol reagent (Invitrogen, Cartsbad, USA). cDNA was synthesized using the GoScript™ Reverse Transcription System (Promega, Madison, USA). Transcription of genes was determined by qPCR using SYBR Green mix (Promega, Madison, USA) with the following PCR conditions: 95 °C for 2 min, followed by 40 cycles of 95 °C for 15 sec and 60 °C for 1 min. β-actin was used as a housekeeping gene for quantification. Relative mRNA copies were compared with negative control using the comparative cycle threshold method (2^-ΔΔCt^). The primer pairs are listed in Supplementary Table 1.

### Western blot analysis

Cell lysates were prepared using Triton X-100 lysis buffer (150 mM NaCl, 20 mM Tris, 1% Triton X-100, 1% sodium deoxycholate, 0.1% sodium dodecyl sulphate (SDS), 10 mM ethylenediaminetetraacetic acid; EDTA) containing cocktail protease inhibitor (Roche, Mannheim, Germany). Protein samples were denatured at 95 °C for 5 min, separated by SDS-polyacrylamide gel electrophoresis and then transferred to polyvinyl difluoride membranes (Millipore, USA). After blocking with 5% albumin bovine (BSA; Amresco, Solon, USA) for 1 h, the membranes were incubated with primary antibodies overnight at 4 °C. After extensive wash in Tris-buffered saline with Tween 20 buffer, the membranes were incubated with horseradish peroxidase-conjugated secondary antibody in 1:3000 dilution (Sino Biological Inc, Beijing, China) for 1 h at room temperature. The signal was detected using an ECL Western blotting detection system (Millipore, USA). Primary antibodies against ANO1 (ab53212, Abcam, USA) and proteins in TNF-α signaling pathway (Cell Signaling Technology, USA) were all diluted to 1:1000. Primary antibody against β-actin (Sino Biological Inc., Beijing, China) was diluted to 1:2000 for use.

### Cell proliferation assay

Cell proliferation was measured by Cell Counting Kit-8 (Dojindo Laboratories, Japan). In brief, 10 μl of the Cell Counting Kit solution was added into each well of a 96-well plate and the cells were further incubated for 2 h at 37 °C before the absorbance was measured at 450 nm using a microplate reader (Thermo Scientific, USA) with a reference wavelength of 650 nm.

### Apoptosis assays

Apoptosis was assessed using Cell Death Detection ELISA kit (Roche, Mannheim, Germany) following the manufacturer’s instructions with inclusion of positive, negative, and background controls. Cell lysates were prepared and added to each well of the 96-well strip provided in the kit before addition of 80 μl of immunoreagent and incubation for 2 h at room temperature with shaking. After cells were washed for three times, 100 μl 2,2'-azino-bis(3-ethylbenzothiazoline-6-sulphonic acid substrate was added to each well and incubated for 10–20 min. Optical absorbance at 405 nm was read on a plate reader and parallel plates with the same treatments were used for cell counting.

Apoptosis was also assessed by flow cytometry. Cells were detached using trypsin (EDTA free), washed with phosphate-buffered saline (PBS) and stained with Annexin V-FITC/PI kit (KeyGen, China). The stained cells were analyzed by flow cytometry (Beckman, USA). The optimal excitation wavelength for detection of Annexin V-FITC was 488 nm with emission maximum at a wavelength of 530 nm. Red fluorescence of PI was measured (Ex = 488 nm, Em ≥ 630 nm). RWPE-1 cells that overexpressed ANO1 were stained with Annexin V-APC/7-ADD kit (KeyGen, China) to suppress fluorescence interference from pIRES2-GFP vector. The optimal excitation wavelength for detection of Annexin V-APC was 633 nm with emission maximum at a wavelength of 660 nm. Red fluorescence of 7-AAD was measured (Ex = 546 nm, Em ≥ 647 nm).

### Immunostaining

PC-3 cells grown in a 24-well plate were fixed with 4% paraformaldehyde for 30 min, washed with PBS for three times. The cells were permeabilized and blocked by incubation in PBS containing 0.2% (v/v) Triton X-100, 1% (w/v) BSA, and 10% (v/v) goat serum for 30 min at room temperature and incubated with primary antibody (TNF-α rabbit mAb, #8184, Cell Signaling, USA) overnight at 4 °C. After washing in PBS, the cells were incubated with Alexa Flour 488 sheep anti-mouse IgG secondary antibody (1:400; Sino Biological Inc, Beijing, China) for 1 h at room temperature. Cells were washed in PBS and the nuclei were stained with Hoechst 33342. The cells were photographed under high-content analysis system (Operetta®, PerkinElmer, USA), and the fluorescence intensity of TNF-α staining was analyzed by Columbus software.

### TNF-α ELISA

TNF-α levels in the supernatant of PC-3 cell culture were detected using human TNF-α Platinum ELISA kit (eBioscience, Vienna, Austria) according to the manufacturers’ instructions. A standard curve was prepared from human TNF-α standard dilutions and human TNF-α sample concentration was determined. Values were normalized to total cell number at the end of experiment.

### Constructions of lentiviral plasmids and preparation of lentivirus

The lentiviral vector pLKO.1 was used to construct ANO1 shRNA1 and control shRNA plasmids. The shRNA oligos were designed according to siRNA sequence. The oligo sequences of ANO1 shRNA1 and control shRNA are listed in Supplementary Table 3. The oligos were annealed and cloned into the AgeIand *Eco*RI sites of pLKO.1 vector. For lentiviral production, 293LTV cells were co-transfected using Lipofectamine 2000 (Invitrogen, Carlsbad, USA) with pLKO.1 shRNA and packaging plasmids. The culture medium was replaced after 16 h of incubation, and supernatants were collected 48 h and 72 h after transfection. Target cells were infected with lentivirus in the presence of 8 μg/ml Polybrene (M&C Gene Technology, Beijing, China).

### Xenograft tumor formation in nude mice

Adult male BALB/c nu/nu mice, 5-week-old, were provided by the Department of Laboratory Animal Sciences, Peking University Health Science Center (Beijing, China). All experimental animal procedures used in this study were conformed to the Guidelines of the Committee of Research and Ethical Issues of Care and Use Committee of Peking University Health Science Center. Animals were housed under a 12-h alternating light/dark cycle with food and water available ad libitum in SPF animal laboratory. The animals were quarantined for 1 week prior to their use in the study.

A total of 2,000,000 of PC-3 cells treated with lentiviral infection of ANO1 shRNA1 or negative control shRNA (NC) were suspended in 0.1 ml saline solution and injected into the right flank of nude mice. Tumor volumes were measured once a week by measuring two perpendicular dimensions with a caliper according to the formula volume = (*a* × *b*^*2*^)/2, where *a* is the largest and *b* is the smallest dimension of the tumor. Body weights were assessed weekly for 5 weeks before tumors were removed and weighed. Finally, tumor tissue samples were quickly frozen in liquid nitrogen and stored at −80 °C for subsequent RNA isolation.

### Statistical analysis

All experiments in vitro were repeated for at least three times, and data were presented as the means ± SEM. Statistical analyses were performed in GraphPad Prism 5 using Student’s *t* test or analysis of variance. The value of *p* *<* 0.05 was considered statistically significant.

## Electronic supplementary material

Supplemental information
