# Supplementary material for: Inhibition of ANO1/TMEM16A induces apoptosis in human prostate carcinoma cells by activating TNF-α signaling
Source: Cell Death Dis. 2018 Jun 13;9(6):703. doi: 10.1038/s41419-018-0735-2 (PMC5999606; doi:10.1038/s41419-018-0735-2)
Supplement: Supplementary file 1 — Supplemental information [file 41419_2018_735_MOESM1_ESM.pdf]

Supplementary information

Inhibition of ANO1/TMEM16A induces apoptosis in human prostate carcinoma cells by activating TNF-α signaling

Yan Song[1], Jian Gao[1], Lizhao Guan[1], Xiaoling Chen[1], Jianjun Gao[2] and KeWei Wang[1,2¶]

[1]Department of Molecular and Cellular Pharmacology, State Key Laboratory of Natural and Biomimetic Drugs, Peking University School of Pharmaceutical Sciences, Beijing 100191; and [2]Department of Pharmacology, Qingdao University School of Pharmacy, Qingdao 266021, China

¶To whom correspondence should be addressed:
KWW (wangkw@bjmu.edu.cn or wangkw@qdu.edu.cn)
Tel: (8610) 82805065
Fax: (8610) 82805065

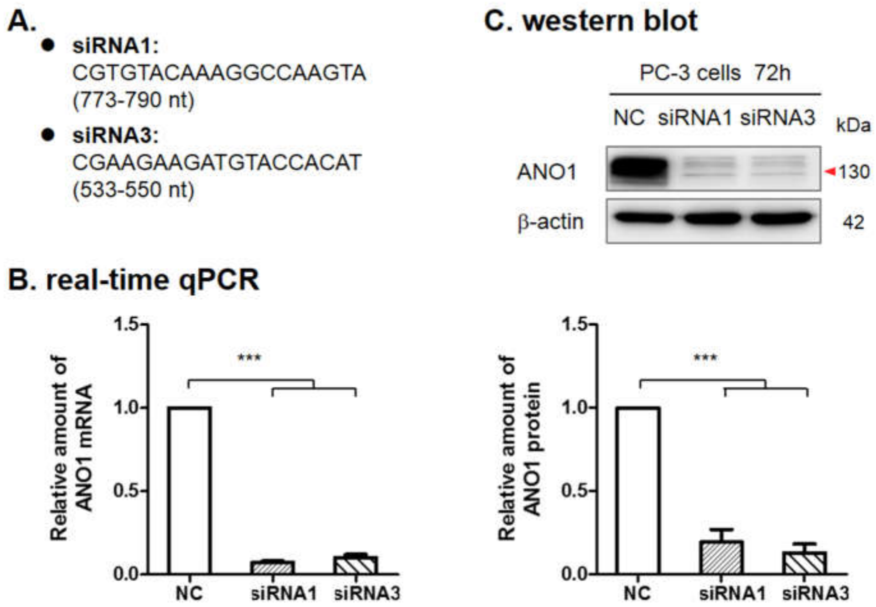

Figure S1. Identification of ANO1 silencing by siRNAs transfection in PC-3 cells.
(A). Two siRNA targeting sites in human ANO1/TMEM16A gene (GenBankNM_018043).
(B). PC-3 cells were transfected by ANO1-siRNAs for 72 h. Relative amount of ANO1 mRNA expression was examined using qPCR and presented after being normalized to β-actin (means ± SEM; $n = 7$ independent experiments). *** $p < 0.001$ are for statistical comparisons.
(C). Top panel, representative images of ANO1 expression by western blot. Bottom panel, quantitative analysis of ANO1 protein expression. Data are expressed as means ± SEM ($n = 5$ independent experiments). *** $p < 0.001$ are for statistical comparisons.

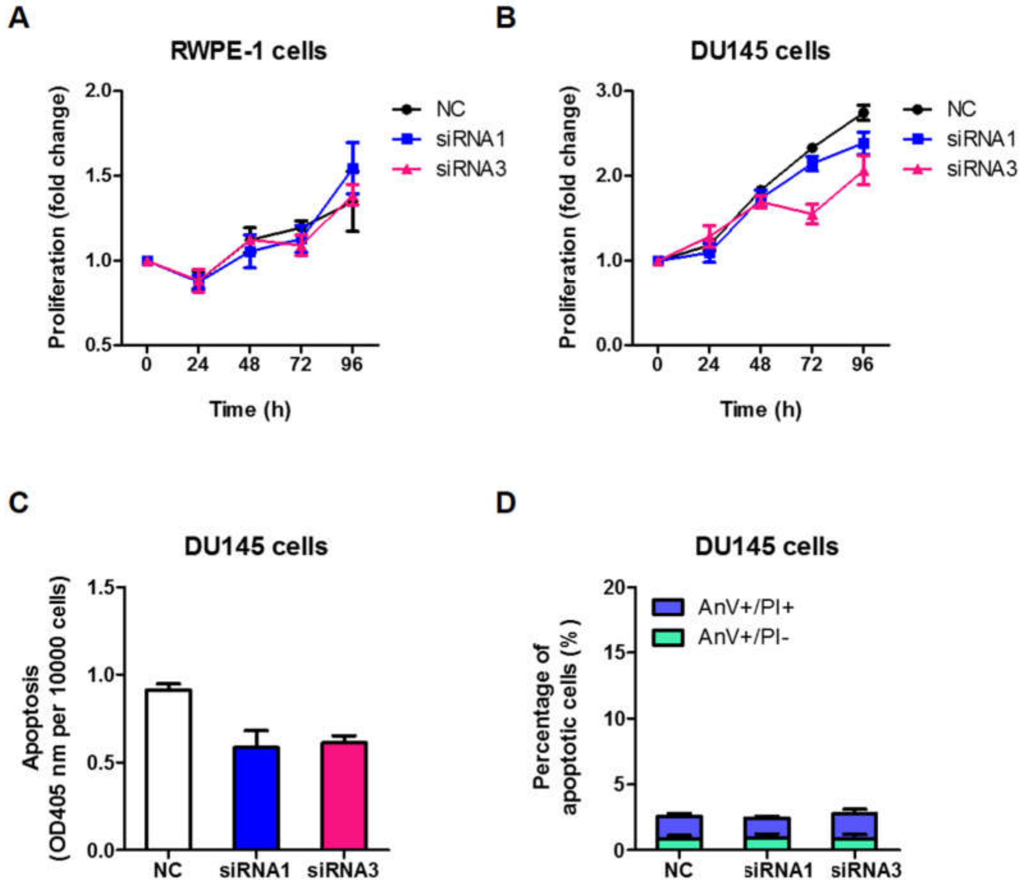

Figure S2. Effect of ANO1 inhibition on cell growth and apoptosis in prostate cancer cells.
(A&B). Silencing of endogenous ANO1 does not significantly decrease cell growth in RWPE-1 or DU145 cells. Cells were transfected with ANO1-siRNAs or NCsi (negative control). Line graph showing relative of viable cell number of RWPE-1 cells (A) or DU145 cells (B) after ANO1 knockdown (means ± SEM, n = 6). Viable cell number was accessed by CCK-8 assay.
(C&D). No effect on apoptosis in DU145 cells by ANO1 silencing. (C) Bar graph showing apoptosis accessed by Cell Death Detection ELISA^{PLUS} Kit (means ± SEM, n = 4). Parallel plates with the same treatment were used for cell counting. (D) Apoptosis was assessed by Annexin V-FITC/PI Apoptosis Detection Kit using a Flow Cytometer. Bar graph showing percentage of apoptotic cells after knockdown of ANO1 for 72 h (means ± SEM, n = 3).

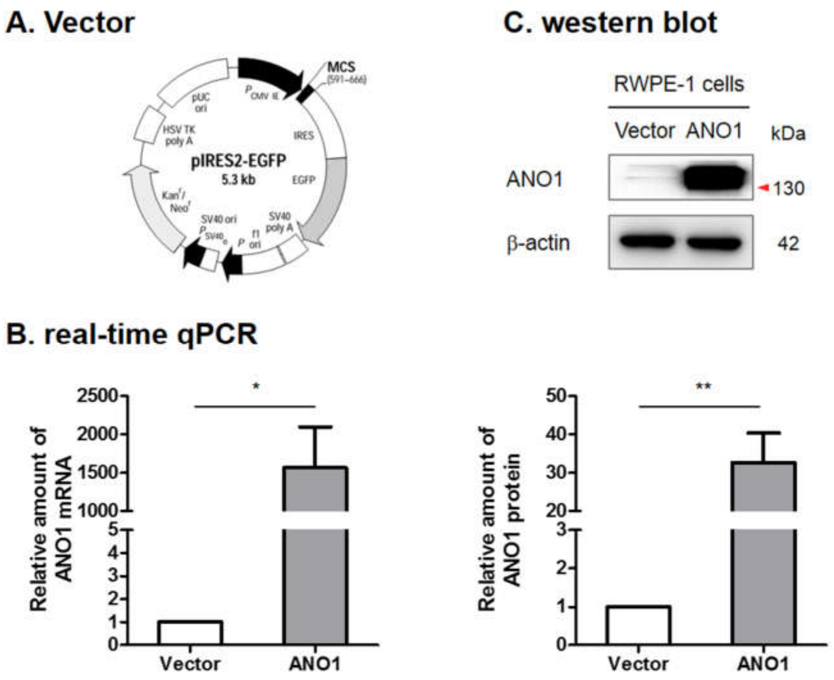

Figure S3. Identification of ANO1 stable overexpression in RWPE-1 cells.

(A). The vector pIRES2-EGFP was used to construct ANO1 plasmids.

(B). Relative amount of ANO1 mRNA expression was examined using qPCR and presented after being normalized to β-actin (means ± SEM; n = 3 independent experiments). * $p < 0.05$ are for statistical comparisons.

(C). Top panel, representative images of ANO1 expression by western blot analysis. Bottom panel, quantitative analysis of ANO1 protein expression. Data are expressed as means ± SEM (n = 4 independent experiments). ** $p < 0.01$ are for statistical comparisons.

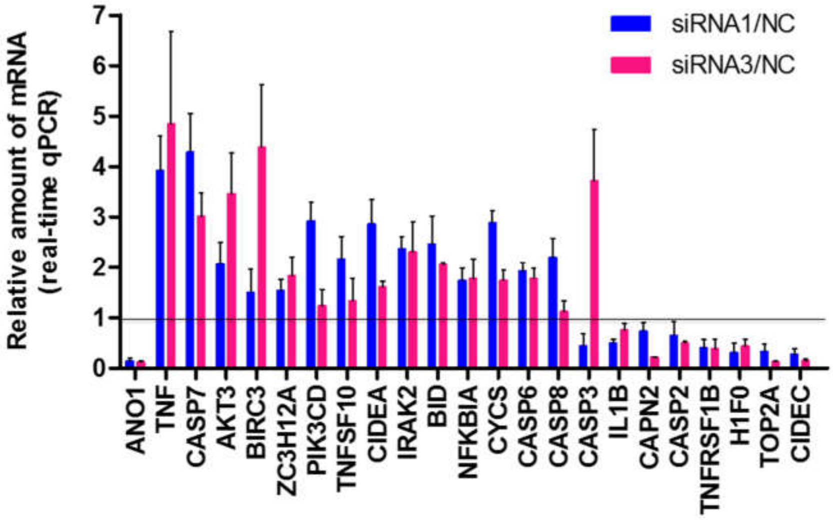

Figure S4. Verification of apoptosis related gene expression in ANO1-RNAi treated PC-3 cell. PC-3 cells were transfected by ANO1-siRNAs for 72 h. Relative amount of ANO1 mRNA expression was examined using qPCR. Data are presented after being normalized to β-actin (means ± SEM; *n* = 3 independent experiments).

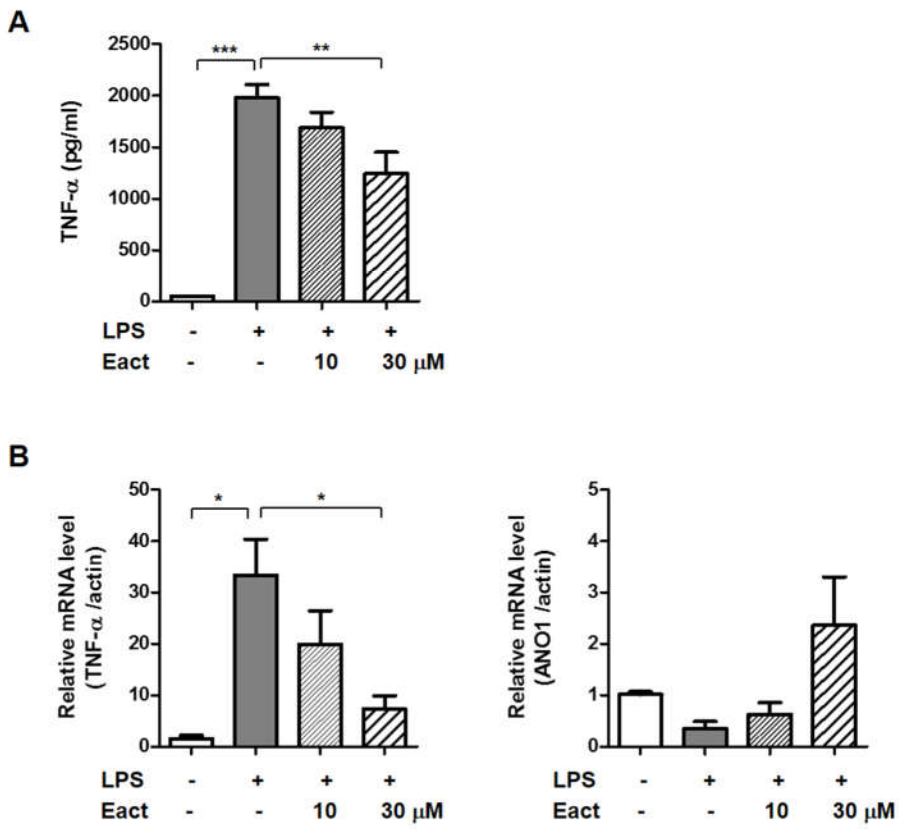

Figure S5. Inhibition of LPS-induced TNF-α production in primary mouse macrophages by pharmacological activation of ANO1.

(A) Primary mouse macrophages were cultured in 24-well plate (1,000,000 cells per well). Cells were pre-treated with ANO1 activator, Eact (10, 30 μM) for 24 h, followed by stimulation with 0.1 μg/mL LPS to induce TNF-α release. After 24 h of LPS stimulation, levels of TNF-α in culture supernatants were measured by ELISA. The data are presented as means ± SEM (n = 9). ** $p < 0.01$, *** $p < 0.001$. Activation of ANO1 by Eact inhibits LPS-induced TNF-α production in a dose-dependent manner.

(B) The relative mRNA expression of TNF-α and ANO1 was examined using qPCR and data are presented after being normalized to β-actin (means ± SEM; n = 4). * $p < 0.05$. LPS up-regulated the mRNA expression of TNF-α, and down-regulated the mRNA expression of ANO1. Eact dose-dependently inhibited the expression of TNF-α induced by LPS-stimulation in primary mouse macrophages.

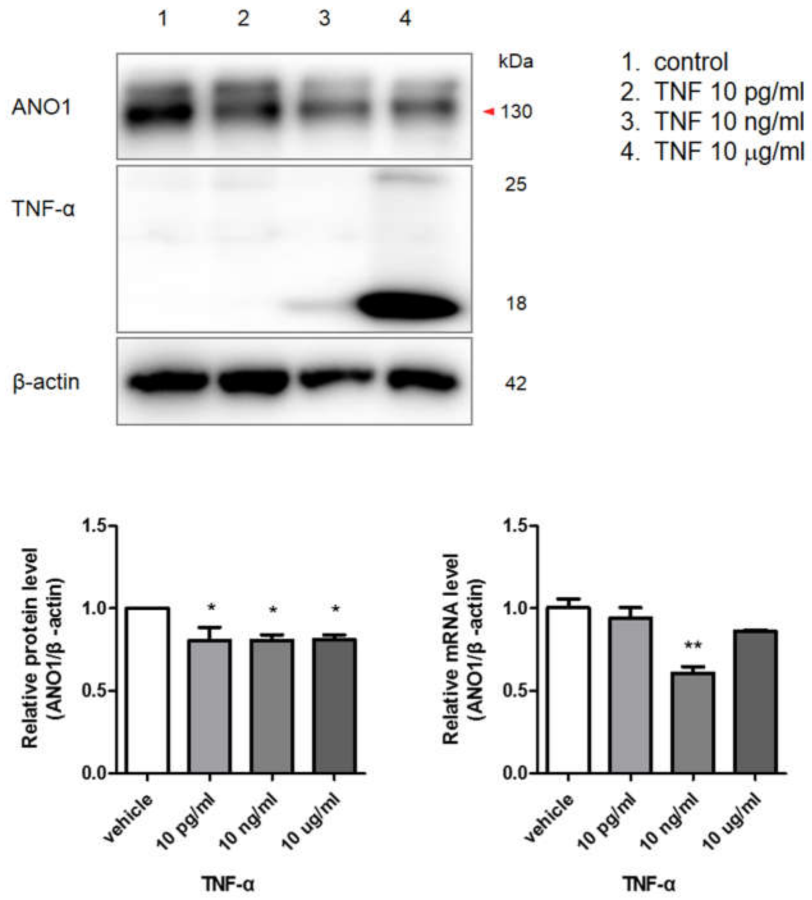

Figure S6. TNF-α down-regulates ANO1 expression in PC-3 cells.

PC-3 cells were cultured in 6-well plate (100,000 cells per well) and were treated with different concentrations of human recombinant TNF-α (10 pg/ml, 10 ng/ml, and 10 μg/ml). After 24 h of treatment, the relative protein and mRNA expression of ANO1 was measured using western blot and qPCR. The data are presented after being normalized to β-actin (means ± SEM; n = 4; * $p < 0.05$, ** $p < 0.01$). Human recombinant TNF-α decreases ANO1 expression in PC-3 cells.

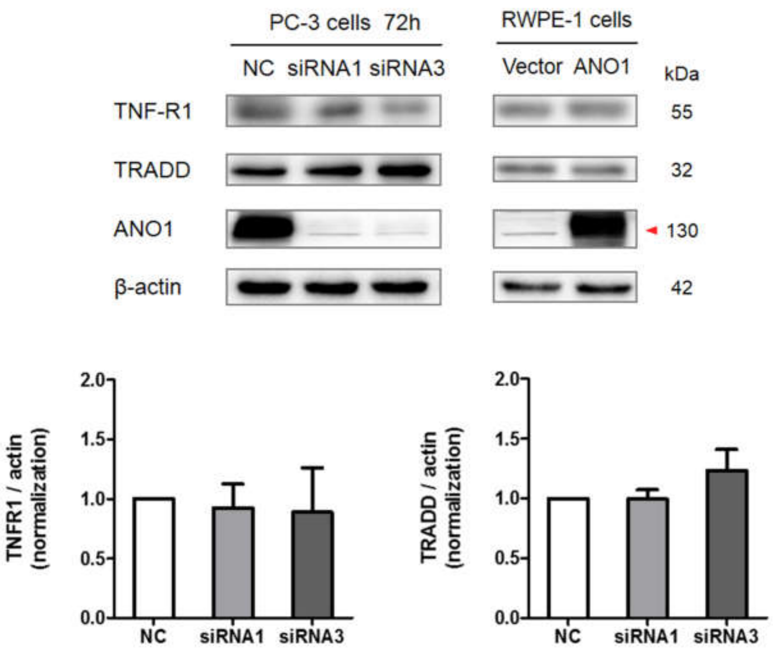

Figure S7. Verification of TNF-R1 and TRADD expression.

Immunoblots of lysates from prostate cells as described. Data are expressed as means ± SEM. There was no significant change observed in TNFR1 (n = 3) and TRADD (n = 5) expression in either PC-3 cells or RWPE-1 cells.

Table S1. Differential expression of apoptosis related genes in ANO1-RNAi treated PC-3 cells by digital gene expression profiling.

Symbol	Description	log_2Ratio (siRNA1/NC)	log_2Ratio (siRNA3/NC)
CASP7	caspase 7	1.461	1.145
AKT3	AKT serine/threonine kinase 3	0.582	1.591
BIRC3	baculoviral IAP repeat containing 3	0.071	2.063
ZC3H12A	zinc finger CCCH-type containing 12A	0.798	1.233
PIK3CD	phosphatidylinositol-4,5-bisphosphate 3-kinase catalytic subunit delta	1.345	0.681
TNFSF10	tumor necrosis factor superfamily member 10, TRAIL	1.183	0.826
CIDEA	cell death-inducing DFFA-like effector a	0.981	0.978
IRAK2	interleukin 1 receptor associated kinase 2	0.678	1.253
BID	BH3 interacting domain death agonist	1.327	0.594
NFKBIA	NFKB inhibitor alpha	0.605	1.304
CYCS	cytochrome c, somatic	1.248	0.558
CASP6	caspase-6	0.883	0.684
CASP8	caspase-8	0.727	0.170
CASP3	caspase-3	-1.540	1.143
IL1B	interleukin 1 beta	-1.344	-0.117
CAPN2	calpain 2	-0.423	-1.533
CASP2	caspase-2	-1.234	-0.823
TNFRSF1B	tumor necrosis factor receptor superfamily member 1B, TNFR2	-0.756	-1.759
H1F0	H1 histone family, member 0	-2.815	-1.985
TOP2A	topoisomerase (DNA) II alpha 170kDa	-2.325	-2.960
CIDEC	cell death inducing DFFA like effector c	-1.926	-3.929

log_2Ratio > 0 means up-regulation, log_2Ratio < 0 means down-regulation

Table S2. Sequences of primers used for qPCR

Gene	Forward (5'…3')	Reverse (5'…3')
hβ-actin	CATGTACGTTGCTATCCAGGC	CTCCTTAATGTCACGCACGAT
hANO1	GAGCCAAAGACATCGGAATCTG	TGAAGGAGATCACGAAGGCAT
hTNF-α	CATGTACGTTGCTATCCAGGC	CCCTAAGCCCCCAATTCTCT
hAKT3	TGTGGATTTACCTTATCCCCTCA	GTTTGGCTTTGGTCGTTCTGT
hBID	ATGGACCGTAGCATCCCTCC	GTAGGTGCGTAGGTTCTGGT
hBIRC3	AAGCTACCTCTCAGCCTACTTT	CCACTGTTTTCTGTACCCGGA
hCAPN2	GTTCTGGCAATACGGCGAGT	CTTCGGCTGAATGCACAAAGA
hCASP2	AGCTGTTGTTGAGCGAATTGT	AGCAAGTTGAGGAGTTCCACA
hCASP3	CATGGAAGCGAATCAATGGACT	CTGTACCAGACCGAGATGTCA
hCASP6	ATGGCGAAGGCAATCACATTT	GTGCTGGTTTCCCCGACAT
hCASP7	CGGTCCTCGTTTGTACCGTC	CGCCCATACCTGTCACTTTATCA
hCASP8	TTTCTGCCTACAGGGTCATGC	GCTGCTTCTCTCTTTGCTGAA
hCIDEA	TTATGGGATCACAGACTAAGCGA	TGCTCCTGTCATGGTTGGAGA
hCIDEC	AAGTCCCTTAGCCTTCTCTACC	CCTTCCTCACGCTTCGATCC
hCYCS	CTTTGGGCGGAAGACAGGTC	TTATTGGCGGCTGTGTAAGAG
hH1F0	CGCGCCAGTCCATTCAGAA	ACAACTTGATCTGCGAGTCAG
hIL1B	ATGATGGCTTATTACAGTGGCAA	GTCGGAGATTCGTAGCTGGA
hIRAK2	GAAATCAGGTGTCCCATTCCAG	TGGGGAGGTCGCTTCTCAA
hNFKBIA	CTCCGAGACTTTCGAGGAAATAC	GCCATTGTAGTTGGTAGCCTTCA
hPIK3CD	AAGGAGGAGAATCAGAGCGTT	GAAGAGCGGCTCATACTGGG
hTNFRSF1B	CGGGCCAACATGCAAAAGTC	CAGATGCGGTTCTGTTCCC
hTNFSF10	GCTCGTTGGTAAAGTACACGTA	TGCGTGCTGATCGTGATCTTC
hTOP2A	ACCATTGCAGCCTGTAAATGA	GGGCGGAGCAAAATATGTTCC
hZC3H12A	GGCAGTGAACTGGTTTCTGGA	GATCCCGTCAGACTCGTAGG

Table S3. Sequences of ANO1 shRNA

Gene	Forward (5'…3')	Reverse (5'…3')
ANO1 shRNA1	CCGGGACGTGTACAAAGGCC AAGTACTCGAGTACTTGGCC TTTGTACACGTCTTTTTG	AATTCAAAAAGACGTGTACA AGGCCAAGTACTCGAGTACT ATGGCCTTTGTACACGTC
control shRNA	CCGGGACGAGTGGTCTAGTT GAGAACTCGAGTTCTCAACT AGACCACTCGTCTTTTTG	AATTCAAAAAGACGAGTGGT CTAGTTGAGAACTCGAGTTC TCAACTAGACCACTCGTC
